# Postoperative Assessment of the Quality of Life in Patients with Colorectal Endometriosis

**DOI:** 10.3390/jcm10215211

**Published:** 2021-11-08

**Authors:** Claudia Mehedintu, Francesca Frincu, Lacramioara Aurelia Brinduse, Andreea Carp-Veliscu, Elvira Bratila, Clotilde Hennetier, Horace Roman

**Affiliations:** 1 Department of Obstetrics and Gynecology, “Carol Davila” University of Medicine and Pharmacy, 020021 Bucharest, Romania; claudiamehedintu@yahoo.com (C.M.); andreea_veliscu@yahoo.com (A.C.-V.); elvirabarbulea@gmail.com (E.B.); 2 Department of Public Health and Management, “Carol Davila” University of Medicine and Pharmacy, 020021 Bucharest, Romania; lbrinduse@gmail.com; 3 Expert Center in the Diagnosis and Multidisciplinary Management of Endometriosis, CHU de Rouen (Charles Nicolle), 76000 Rouen, France; clotilde.hennetier@chu-rouen.fr; 4 Endometriosis Center, Clinique Tivoli-Ducos, 33000 Bordeaux, France; horace.roman@gmail.com; 5 Department of Gynecology and Obstetrics, Aarhus University Hospital, 8200 Aarhus, Denmark

**Keywords:** HRQoL, SF-36, GIQLI, KESS, DE, colorectal endometriosis

## Abstract

Morbidity and mortality alone are not comprehensive measures of evaluating the benefits of surgical interventions in endometriosis patients, thus, subjective patient-reported instruments are required. The 36-tem Short Form Survey (SF-36) is a Health-Related Quality of Life (HRQoL) instrument that has not been validated yet for women with endometriosis. The aims of this study are to evaluate the validity and reliability of the SF-36 in patients with colorectal endometriosis and to compare the HRQoL before and after surgery, using different Quality of Life (QoL) instruments: the Gastrointestinal QoL Index (GIQLI) and Knowles–Eccersley–Scott Symptom Questionnaire (KESS). We conducted a retrospective study using prospectively recorded data in the North-West Inter-Regional Female Cohort for Patients with Endometriosis (CIRENDO) database. The assessment was performed on four hundred and eighty-eight patients before and 12 months after the surgery. Preoperative and postoperative item-internal consistency and Cronbach’s α proved evidence for good reliability showing that SF-36 is a useful instrument for endometriosis patients’ QoL. The domains of Role (limitation) physical, Bodily pain and Role (limitation) emotional showed the most remarkable improvements (difference before vs. one year after surgery) with *p* < 0.001. Our data show that SF-36 has validity and reliability and can be used in patients with endometriosis. Surgery improved the QoL and digestive function.

## 1. Introduction

Deep endometriosis (DE) is characterized by the presence of endometriosis lesions that infiltrate the peritoneum by >5 mm [1]. It occurs in approximately 1% of women of reproductive age and it affects between 4% and 37% of women with pelvic endometriosis [2]. The DE lesions typically affect the Douglas pouch, the uterosacral ligaments, the posterior vaginal wall, the anterior rectal wall, the sigmoid colon, the rectum and the urinary tract [3]. Between 90 and 95% of women diagnosed with DE experience severe pain, most commonly in the form of dysmenorrhea, deep dyspareunia and intermenstrual pelvic pain [4]. Additional gastrointestinal symptoms depend on the affected area and raise the clinical suspicion for infiltrative endometriosis lesions of the bowel. When the rectum and the sigmoid are affected, symptoms include deep dyspareunia, dyschezia, rectorrhagia, catamenial diarrhea and narrowed stools. Surgical management of colorectal DE is required when the lesions become symptomatic and impair bowel, reproductive and sexual functions [5]. 

Endometriosis affects the quality of life (QoL) of young, active women by disrupting their ability to work, their sexual functioning and their social relationships [6]. The HRQoL (Health-Related Quality of Life) is a multidimensional concept and reflects how a patient perceives their own physical and mental state. This perception is shaped not only by the disease itself but also by the impact of the disease on the social and family life of the patient. As a result, morbidity and mortality alone are not comprehensive measures of evaluating the benefits of surgical interventions in endometriosis patients and subjective, patient-reported instruments are required. The 36-Item Short Form Survey (SF-36) is a well-established HRQoL instrument that hasn’t been validated yet for women with endometriosis. It includes eight distinct health status domains, with a high level of comprehensiveness. Higher scores indicate a better health status [7]. 

The aim of this study is to evaluate the validity and reliability of SF-36 in patients with endometriosis before and after surgical treatment. A second objective is to compare the HRQoL pre and postoperatively, using different instruments apart from SF-36, such as the Gastrointestinal Quality of Life Index (GIQLI) and Knowles–Eccersley–Scott Symptom (KESS) questionnaire, which are validated and suitable for endometriosis-related gastrointestinal symptoms and the Visual Analogue Scale (VAS), as shown by Bourdel N. et al. in an exhaustive systematic review, which is considered the “gold-standard” for pain-measurement [8].

## 2. Materials and Methods

The present study is a retrospective analysis of prospectively recorded data from June 2009 to November 2016 at Rouen University Hospital, Rouen, France and includes patients with surgical procedures for DE nodules of the rectum or sigmoid colon. The procedures, both laparoscopies and laparotomies, were performed by our team, which has significant experience in the surgical management of colorectal endometriosis. The women were enrolled in the CIRENDO database (the North-West Inter-Regional Female Cohort for Patients with Endometriosis), a prospective cohort with financial support by the G4 Group (The University Hospitals of Rouen, Lille, Amiens, and Caen). Prospective data recording and analysis were approved by the French authorities CNIL (Commission Nationale de l’Informatique et des Libertés: the French data protection commission) and CCTIRS (Comité Consultatif pour le Traitement de l’Information en matière de Recherche dans le domaine de la Santé: the advisory committee on information technology in healthcare research, NCT02294825). 

Of the patients enrolled in CIRENDO, we selected for analysis those with histologically confirmed endometriosis and one-year follow-up visit. The surgical and histological records, as well as the self-assessment questionnaires completed before and after the surgery, provide information for the study. Data were recorded by a clinical researcher in charge of evaluating patients’ follow-up. 

All patients filled in baseline standardized questionnaires, i.e., SF-36, GIQLI, KESS, before surgery and one year after, as implemented by the CIRENDO protocol. The Visual Analog Scale (VAS) was also used for the evaluation of dysmenorrhea and dyspareunia, with and without pain relief medication. The French-language versions of the SF-36, GIQLI and KESS questionnaires were validated long ago on large cohorts [8].

The SF-36 self-administered questionnaire incorporates 36 standardized items evaluating the generic health status of a patient [7]. This survey evaluates 8 health domains: physical functioning (PF); role limitations due to physical problems (RP); bodily pain (BP); general health perception (GH); vitality (VT); social functioning (SF); role limitation due to emotional problems (RE); mental health (MH). The Physical and Mental Component Summary scores (PCS and MCS) represent the composite groups based on the typical factor structures that are hypothesized in SF-36, as follows: PF, RP, BP and GH as being domains of the PCS and RE, VT, MH and SF as being domains of the MCS. Each of the 8 domains has a score that ranges from 0 to 100. Higher scores indicate a better health status [9]. 

The GIQLI and KESS questionnaires are used when describing, comparing and differentiating the outcomes of surgical treatment in patients with gastrointestinal diseases. The GIQLI survey consists of 36 questions targeting intestinal dysfunctions, with scores ranging from 0 (worst) to 144 (best) [10]. The KESS survey is an 11-item tool that measures various degrees of constipation. A KESS score can range from 0 (no symptoms) to 39 (high symptom severity), with a cut-off score ≥ 11 that indicates the presence of constipation [11]. The VAS ranges from 0 to 10, with its extremes marked as “no pain” and “worst imaginable pain” [8]. 

The baseline and management characteristics and questionnaire results before and after surgery are presented as absolute and relative frequencies for qualitative variables and as mean and standard deviations for quantitative variables. 

Two techniques were used for the evaluation of the internal consistency of the SF-36 questionnaire: item-scale correlation and Cronbach’s α. The item-scale correlation was assessed by using item-internal consistency and item-discriminant validity. Item-internal consistency estimates the correlation between the implied item and the sum of the other items on the same domain; its value must be greater than 0.4 [12]. Item-discriminant validity was evaluated using the scaling success rate (%) for each scale. A scaling success rate was calculated for each question of SF-36, as the percentage of item-own scale correlations that are significantly larger than the correlations between the item and the other concurrent scales [13]. Cronbach’s α globally measures the correlations between the items of a scale. The reliability of a questionnaire is considered acceptable when Cronbach’s α value is greater than 0.7 [14]. Both item-scale correlation and Cronbach’s α were analyzed pre and postoperatively. 

Construct validity was evaluated by comparing the SF-36 scores and pain (dysmenorrhea and dyspareunia, with and without treatment) intensity levels, both pre and postoperatively. The Pearson correlation coefficient was used for evaluating the relationship between HRQoLs as measured by the SF-36 and pain intensity as measured by the VAS.

A paired T-test of differences before and one year after surgery was used to determine changes in the SF-36 (evaluating HRQoL), the KESS and GIQLI scores and in the pain intensity VAS score. Observed differences between pre-op and one-year post-op values were described using 95% confidence intervals (95% CI). For the patients whose KESS and GIQLI scores showed a worsening of digestive symptoms, the evaluation of the HRQoL was compared separately using a T-test for means comparison. 

Statistical analysis was performed using the software IBM SPSS Statistics for Windows, Version 23.0. Armonk, NY, USA: IBM Corp.

## 3. Results

We enrolled 488 patients from the CIRENDO database. Patients’ characteristics at baseline are presented below in Table 1. Of them, 56.6% had a history of gynecologic surgery. The mean revised American Fertility Society classification (AFS-r) score was 69.6 ± 41.4. Among the patients included in the study, 29.1% reported a history of mental health disorders (depression, anxiety, sleep disorders, panic attacks), 97.7% had dysmenorrhea and 76.2% had dyspareunia. 

The most important intraoperative findings and surgical procedures are represented below. Postoperative complications were reported in the order of their appearance, as follows: ureteral fistula 7%, digestive tract fistula 2.2%, pelvic abscess (surgical management) 0.6%, bladder atony 0.6%, pelvic abscess (conservative management) 0.4% and others 1.8% (Table 2).

Dysmenorrhea intensity, as measured by the VAS, both with and without pain relief therapy (PRT), which primarily consisted of nonsteroidal anti-inflammatory drugs (NSAIDs), decreased significantly (*p* < 0.001) at one year post-surgery compared to preoperative evaluations. This intensity decreased from 8.4 ± 1.5 to 2.1 ± 3.2 points without PRT and from 4.7 ± 2.3 to 2.9 ± 2.1 with PRT. A similar evolution was noticed in dyspareunia intensity without PRT. We observed a significant decrease in VAS score (*p* < 0.001) from 5.6 ± 2.4 preoperative to 1.9 ± 2.8 post-op at one year. Dyspareunia with PRT only slightly decreased after surgery, from 4.0 ± 2.3 to 3.8 ± 2.3, without achieving statistical significance (*p* = 0.657) (Table 3).

Item-internal consistency exhibited preoperative values of 0.472 (General Health domain), 0.760 (Bodily Pain dimension) and signaled good internal consistency. Item-internal consistency had better postoperative values for all of the eight domains of the SF-36.

Scaling success rates were used to evaluate item-discriminant validity. The preoperative scaling success rate had values greater than 98.2%. The postoperative scaling success rate showed values greater than 99% for 7 of the 8 domains of SF-36. The values of this reflected higher correlations with their hypothesis scales than with other scales, both pre and postoperatively, except the Vitality domain, which slightly decreased in postoperative, from 98.2% to 97.7%, due to high correlation between the vitality item (VT) and distinct scales.

Internal consistency measured with Cronbach’s α was greater than 0.7 for all of the eight domains of the SF-36, both pre- and post-op. Cronbach’s α for pre and postoperative PF and for postoperative BP exceeded the 0.9 threshold, meeting the recommended standard criteria for individual comparisons (Table 4).

All of the SF-36 domains and the summary scales (physical and mental component scores) revealed significantly higher mean values postoperatively (*p* < 0.001), than the preoperative mean scores. The most remarkable improvements were observed in the domains of Role (limitation) physical (26.9), Bodily pain (25.4) and Role (limitation) emotional (20.8).

The Vitality, Mental Health and General Health domains showed a weaker improvement of HRQoL (10.2, 12.0 and 12.3, respectively). All of the SF-36 domains scored inferior values for the HRQoL compared to a nationally representative sample of healthy French women aged 25–34, both preoperatively and postoperatively (Table 5).

All SF-36 domains correlated negatively with dysmenorrhea (with and without treatment) and dyspareunia (without treatment), as measured by the VAS, both preoperatively and postoperatively. The scores for dyspareunia with treatment were significantly correlated with the preoperatively Bodily Pain scores and with the postoperatively General Health and Social Functioning scores (Table 6).

Both KESS and GIQLI scores significantly improved one year after surgery (*p* < 0.001). The KESS score dropped from 10.6 ± 7.6 to 9.1 ± 7.1 and the GIQLI score increased from 76.6 ± 35.9 to 90.5 ± 38.5 (Table 3). The KESS score improved in 50.8% of the patients, worsened in 30.5% and remained stationary for the rest. In patients whose KESS score worsened, the scores for the eight domains of the HRQoL significantly improved; however, they did so to a lesser extent compared to the whole cohort of the study. The smallest increases were recorded for the Vitality (5.2), General Health (6.4) and Mental Health (8.5) domains (Table 7).

The GIQLI score improved in 66.8%, worsened in 21.3% and remained stationary in 11.9% of the patients. The 104 patients whose GIQLI score decreased also experienced a decrease in the scores for most HRQoL domains but without statistical significance. Bodily Pain is the only SF-36 domain that recorded a statistically significant increase (*p* = 0.006), in addition to the Physical component score (*p* = 0.047). Patients with worsened GIQLI scores also experienced higher scores for the Physical Functioning and Role (limitation) physical domains (*p* = 0.562 and *p* = 0.104, respectively), without statistical significance.

## 4. Discussion

Our study reports data from multiple questionnaires in order to assess the HRQoL in women with rectal and sigmoid DE, before and one year after surgical excision. All questionnaires that were administered showed mostly significantly higher HRQoL scores at one-year follow-up compared with the pre-op scores. Though some patients did not show improvement on several items on the questionnaires, the overall HRQoL appears to have increased. As in other studies, we concluded that surgery for DE involving the colon and the rectum generally improves a patients’ HRQoL. Our study demonstrates that score variations are concordant. These variations also seem suitable for assessing different dimensions of HRQoL: pelvic comfort, emotions, physical well-being, gastrointestinal function, etc.

Our study has several strengths. It is based on prospective data collected from a large cohort of 488 patients whose follow-up was monitored by a clinical researcher. Multiple questionnaires were used before and one year after surgery in order to assess the HRQoL and digestive function of the patients and to provide an accurate and comprehensive evaluation of the effect of surgical excision for colorectal endometriosis. The SF-36 questionnaire also allowed for a comparison with French normative data. This comparison underlined a higher HRQoL in treated patients, in contrast to patients who still suffer from colorectal DE.

One weakness of our study is that we do not provide an interpretation of the variation in the scores depending on postoperative complications, recurrences or sequelae. However, as mentioned above, the main objective of our study was to evaluate the validity and reliability of the SF-36 questionnaire in patients with colorectal DE. Moreover, an additional objective was using various tools in order to compare pre and postoperative HRQoL.

The SF-36 questionnaire has been validated in many studies that have established its importance in the setting of various pathologies [16,17]. As mentioned above, internal consistency measured with Cronbach’s α is greater than 0.7 for all eight domains, both pre and postoperatively. This translates into a good degree of validity and reliability for the SF-36 questionnaire. The cardinal advantages are represented by its self-administered form, the large addressability and high level of comprehensiveness. This generic tool is suitable for evaluating the HRQoL of patients in different stages of endometriosis. Moreover, SF-36 has the ability to point out different characteristics in a certain population (for example, the pain intensity that affects mental health) and it allows for comparisons with the general population [17].

Redwine and Wright looked at the differences in the postoperative SF-36 scores across patients with both major and slight improvements in their HRQoL. They predicted a good response to surgery when spontaneous pain during physical examination correlated with Douglas pouch obliteration through a palpable nodule [18]. Dubernard et al. further developed the idea of prediction for surgical responses and elaborated a mathematical model for women with symptomatic endometriosis that predicts improvement in their SF-36 scores. The probability of HRQoL improvement after surgery was 80.7% when preoperative Physical Component Summary (PCS) scores were below 37.5. The probability of HRQoL improvement after surgery was 84.2% when preoperative Mental Component Summary (MCS) scores were below 44.5 [19]. Similarly, mean values of PCS and MCS increased after surgery from 50.9 to 70.4 and from 45.3 to 60.6, respectively. This suggests, in turn, a significant improvement in HRQoL.

The domains that exhibited the largest improvement 12 months after surgery were Role Limitation (RP) (physical), Role Limitation (RE) (emotional), and Bodily Pain (BP). These domains exhibited some of the lowest preoperative scores (all below 50). Our results are in line with most of the other studies [20,21,22]. Ribeiro et al. reported increased mean values for the BP domain from 25.83 to 79.56 [20]. A recent randomized study conducted by our team, which compared radical and conservative rectal surgery, used SF-36 to demonstrate the improvement in HRQoL after both conservative surgery and segmental resection. The results appear to be similar across these surgical techniques and they consist of positive outcomes at the clinical assessment 24 months after the surgery. The RP domain showed excellent values of 100, both in the conservative and the radical groups, while the BP domain revealed slightly better results in the conservative group (84, compared to 74 in the radical group). The RE domain revealed mean values of 100 as well [23]. In the current study, the mean values for the RP and the RE domains also increased. Garavaglia et al. showed an overall improvement in all of the SF-36 domains for operated patients for colorectal endometriosis, with consistent increases for the physical role, emotional role and pain subscales (*p* = 0.0001; *p* = 0.001; *p* = 0.003, respectively) [24]. Another recent study conducted by Marlon de Freitas Fonseca et al. showed that all of the patients with dysmenorrhea and chronic pelvic pain experienced an improvement in all of the SF-36 subscales after laparoscopic surgery for endometriosis and concluded that “the more intense the pain, the worse the quality of life” [25]. Meuleman et al. repeatedly stated in extensive reviews that, in general, most studies showed significant improvement in HRQoL after surgery [26,27].

It can be challenging for patients to deal with gastrointestinal symptoms due to colorectal endometriosis or surgical treatment procedures [28]. In our study, both the KESS and the GIQLI scores showed improvement. Our previous studies have reported similar findings [29,30]. One of these pointed out that the worst KESS scores in untreated women were registered in DE of the rectum (with a value of 13.1) compared to cases of DE without rectum involvement (with a value of 10.4) [31]. Previous KESS and GIQLI scores suggest that better digestive function results are achieved when using conservative techniques such as shaving or disc excision because of the ability to avoid low anterior resection syndrome [5,32]. Some of the patients did not show any improvement in the digestive symptoms and expressed worsening of the KESS and GIQLI scores. However, the analysis of the SF-36 results in this paper suggests that HRQoL was better after surgery. Most of our studies have reported similar findings, with a focus on the constipation subscale from the GIQLI score, which did not suffer any change [29,30,31,33].

Previous studies on the topic mainly emphasize the improvement in pain after laparoscopic surgery for colorectal DE [34,35]. In this regard, our findings showed significant improvement in dysmenorrhea one year after surgery. This was quantitatively illustrated with the results from the VAS and regardless of the association with pain relief therapy (PRT) (*p* < 0.001). Therefore, pain intensity in the form of either dysmenorrhea or dyspareunia, with or without treatment, represents an important component of diminished self-perceived HRQoL. In a prospective study on patients with colorectal excision for endometriosis, Bailly et al. showed significant improvement in postoperative VAS scores for dysmenorrhea (*p* < 0.0039), dyspareunia (*p* < 0.0084), chronic pelvic pain (*p* < 0.0002) and dyschezia (*p* < 0.0312) [35]. Our results also show improvement in dyspareunia symptoms, but only in the group without additional PRT. In a prospective cohort study on HRQoL after colorectal resection for endometriosis, Abbot et al. reported similar results to ours. Dyspareunia and dyschezia VAS median scores showed improvement at one-year follow-up, compared to the baseline (66 to 5 and 48 to 20, respectively) but did not reach statistical significance (*p* < 0.080 and *p* < 0.173, respectively). Hence, the same study proved that dysmenorrhea and chronic pelvic pain VAS median scores improved one year after surgery (71 to 5 and 74 to 11, respectively) and reached statistical significance (*p* < 0.028 and *p* < 0.046, respectively) [36].

## 5. Conclusions

Our study corroborates the findings of most of the literature. SF-36 is a valid and reliable survey instrument. It confirms the improvement in the overall HRQoL in women who undergo surgery for colorectal DE. It is of note that surgery also improved HRQoL in patients without objective improvement in digestive function. Our results confirm that surgically-treated colorectal endometriosis associates with better HRQoL and improvement in digestive function, as evaluated with SF-36, GIQLI, KESS and VAS questionnaires. The decision to perform a surgical procedure should be a consideration in symptomatic patients with colorectal DE.

## Figures and Tables

**Table 1:** Patients’ characteristics at baseline (N = 488).

Characteristic	N = 488
**Age** (years, mean ± SD)	32.9 ± 6.5
**BMI (Body Mass Index)**	**N (%)**
≤24.9 kg/m^2^	345 (70.7%)
25.0–29.9 kg/m^2^	96 (19.7%)
≥30 kg/m^2^	47 (9.6%)
**Smoker N (%)**	150 (30.7%)
**History of gynecological surgical procedures N (%)**	276 (56.6%)
Laparotomy	41 (14.8%)
Laparoscopy	212 (76.8%)
Both	23 (8.3%)
**Self-reported mental issues**	142 (29.1%)
Anxiety	106 (21.7%)
Depression	49 (10.0%)
Sleep disorders	79 (16.2%)
Panic attacks	33 (6.8%)
**Dysmenorrhea**	477 (97.7%)
**Dyspareunia**	372 (76.2%)
**AFS-r Score** (The revised American Fertility Society classification of endometriosis) (mean ± SD)	69.6 ± 41.4

**Table 2:** Intraoperative findings and surgical procedures.

Intraoperative Findings and Surgical Procedures	
**Operative route**	
Laparoscopic	440 (90.1%)
Laparotomic	8 (1.6%)
Laparoscopic and laparotomic	29 (5.9%)
**Douglas obliteration**	
Partially	151 (30.9%)
Totally	275 (56.3%)
**Adenomyosis**	60 (12.3%)
**Adhesiolysis**	451 (92.4%)
Right adnexa adhesiolysis	310 (63.5%)
Left adnexa adhesiolysis	350 (71.7%)
Rectovaginal adhesiolysis	416 (85.2%)
**Right endometrioma**	253 (51.8%)
**Left endometrioma**	276 (56.55%)
**Endometriosis nodules**	
Left uterosacral ligament (L us)	81 (16.6%)
Right uterosacral ligament (R us)	58 (11.8%)
Rectovaginal space (RV)	202 (41.4%)
L us + R us + RV	215 (44%)
Ileal lesions	31 (6.3%)
Lesions of the sigmoid	239 (49%)
Lesions of the rectum	429 (88%)
Lesions of the appendix	37 (7.5%)
**Hysterectomy**	78 (15.9%)
**Procedure performed on the digestive tract**	408 (83.6%)
Shaving	163 (33.4%)
Full thickness disk excision	55 (11.3%)
Colorectal segmental resection	122 (25%)
**Temporary stoma**	
Ileostomy	14 (2.8%)
Colostomy	55 (11.2%)
**Procedure performed on the urinary tract**	73 (15%)

**Table 3:** Preoperative and postoperative KESS, GIQLI and VAS scores.

Symptoms	Preoperative	Postoperative (1 Year after Surgery)	*p* Value *
**KESS score (mean, SD)**	10.6 ± 7.6	9.1 ± 7.1	<0.001
**GIQLI score (mean, SD)**	76.6 ± 35.9	90.5 ± 38.5	<0.001
**VAS score (mean, SD)**			
Dysmenorrhea			
No PRT	8.4 ± 1.5	2.1 ± 3.2	<0.001
With PRT	4.7 ± 2.3	2.9 ± 2.1	<0.001
Dyspareunia			
No PRT	5.6 ± 2.4	1.9 ± 2.8	<0.001
With PRT	4.0 ± 2.3	3.8 ± 2.3	0.657

Legend: PRT = pain relief therapy, * *p* value of paired T-test comparing scores before and after surgery.

**Table 4:** Reliability tests.

SF-36 Domains	Number of Items	Preoperative			Postoperative		
		Item-Internal Consistency (MEAN)	Cronbach’s α	Scaling Success Rate (%)	Item-Internal Consistency	Cronbach’s α	Scaling Success Rate (%)
Physical functioning	10	0.549	0.922	99.4	0.562	0.921	99.8
Role (limitation) physical	4	0.497	0.797	99.4	0.687	0.898	99.4
Role (limitation) emotional	3	0.499	0.75	98.6	0.599	0.818	99.6
Vitality	4	0.586	0.85	98.8	0.614	0.863	97.7
Mental health	5	0.563	0.865	98.2	0.619	0.89	99
Social functioning	2	0.709	0.822	99.4	0.754	0.859	99.6
Bodily pain	2	0.76	0.85	99.2	0.836	0.91	99.8
General health	5	0.472	0.812	99.4	0.54	0.848	99.6

**Table 5:** Short Form-36 quality of life scores before and one year after surgery.

Variable	Score before Surgery (Mean ± SD) *	Score One Year after Surgery (Mean ± SD) *	French Normative Data [15] (Mean ± SD) *	Difference (Before vs. One Year after Surgery), Mean (95% CI) **	*p* Value
**Domain**					
Physical functioning	69.8 ± 25.6	82.8 ± 22.1	92.4 ± 14.2	13.1 (10.6–15.4)	<0.001
Role (limitation) physical	41.5 ± 38.0	68.4 ± 40.4	86.0 ± 28.3	26.9 (22.8–31.1)	<0.001
Bodily pain	44.4 ± 26.2	69.8 ± 26.7	80.2 ± 21.3	25.4 (22.6–28.3)	<0.001
General health	48.1 ± 19.1	60.4 ± 21.0	75.1 ± 16.7	12.3 (10.7–14.0)	<0.001
Vitality	35.6 ± 19.4	45.8 ± 21.5	60.6 ± 18.3	10.2 (8.3–12.1)	<0.001
Social functioning	51.9 ± 23.6	70.4 ± 25.4	82.8 ± 20.6	18.5 (15.9–20.8)	<0.001
Role (limitation) emotional	47.7 ± 39.8	68.5 ± 39.2	86.1 ± 28.2	20.8 (16.6–25.0)	<0.001
Mental health	45.9 ± 20.0	57.9 ± 21.6	68.2 ± 18.1	12.0 (10.0–13.9)	<0.001
**Summary scales**					
Physical component score	50.9 ± 21.1	70.4 ± 23.0	83.4 ± 20.2	19.5 (17.3–21.6)	<0.001
Mental component score	45.3 ± 21.6	60.6 ± 23.2	74.4 ± 21.3	15.3 (13.2–17.5)	<0.001

* Mean and SD values are shown for score; ** Mean (95% CI) is shown for differences. French women aged 25 to 34 years were included. Paired *t*-test of differences before and one year after surgery was used.

**Table 6:** Pearson correlations between SF-36 subscale and component scores and dysmenorrhea/dyspareunia VAS, before and one year after surgery.

	Dysmenorrhea				Dyspareunia			
	without Treatment		with Treatment		without Treatment		with Treatment	
	N	r	N	r	N	r	N	r
**Before surgery**								
Physical functioning	477	−0.291**	457	−0.215 **	372	−0.145 **	97	−0.046
Role (limitation) physical	477	−0.298 **	457	−0.249 **	372	−0.115 *	97	0.052
Bodily pain	477	−0.269 **	457	−0.273 **	372	−0.186 **	97	−0210 *
General health	477	−0.263 **	457	−0.242 **	372	−0.199 **	97	−0.011
Vitality	477	−0.256 **	457	−0.248 **	372	−0.217 **	97	−0.071
Social functioning	477	−0.277 **	457	−0.255 **	372	−0.151 **	97	−0.071
Role (limitation) emotional	477	−0.235 **	457	−0.242 **	372	−0.127 *	97	−0.026
Mental health	477	−0.228 **	457	−0.211 **	372	−0.201 **	97	−0.088
Physical component score	477	−0.368 **	457	−0.321 **	372	−0.198 **	97	−0.058
Mental component score	477	−0.294 **	457	−0.287 **	372	−0.196 **	97	−0.069
**One year after surgery**								
Physical functioning	177	−0.343 **	144	−0.477 **	184	−0.198 **	42	−0.111
Role (limitation) physical	177	−0.417 **	144	−0.479 **	184	−0.251 **	42	−0.269
Bodily pain	177	−0.520 **	144	−0.487 **	184	−0.316 **	42	−0.186
General health	177	−0.425 **	144	−0.514 **	184	−0.305 **	42	−0.371 *
Vitality	177	−0.426 **	144	−0.502 **	184	−0.220 **	42	−0.095
Social functioning	177	−0.387 **	144	−0.454 **	184	−0.337 **	42	−0.333 *
Role (limitation) emotional	177	−0.353 **	144	−0.334 **	184	−0.227 **	42	−0.185
Mental health	177	−0.285 **	144	−0.358 **	184	−0.285 **	42	−0.18
Physical component score	177	−0.514 **	144	−0.584 **	184	−0.317 **	42	−0.273
Mental component score	177	−0.420 **	144	−0.461 **	184	−0.302 **	42	−0.227

* *p* < 0.05; ** *p* < 0.001.

**Table 7:** Patients with worsened KESS score, but with better QoL one year after surgery.

Variable	Score before Surgery (Mean ± SD)	Score One Year after Surgery (Mean ± SD)	Difference (Before vs. One Year after Surgery), Mean (95% CI)	*p* Value
N = 149				
**Domain**				
Physical functioning	57.7 ± 20.6	78.5 ± 24.3	25.8 (21.7–29.8)	<0.001
Role (limitation) physical	42.8 ± 38.5	61.7 ± 41.6	18.9 (11.4–26.5)	<0.001
Bodily pain	47.3 ± 26.1	65.7 ± 27.3	18.4 (13.1–23.7)	<0.001
General health	49.5 ± 18.8	55.9 ± 20.8	6.4 (3.6–9.3)	<0.001
Vitality	38.2 ± 19.3	43.4 ± 21.0	5.2 (1.9–8.4)	0.002
Social functioning	55.2 ± 23.2	68.5 ± 25.4	13.3 (9.2–17.5)	<0.001
Role (limitation) emotional	52.8 ± 38.9	68.9 ± 39.6	16.1 (8.6–23.5)	<0.001
Mental health	48.5 ± 20.1	57.0 ± 21.1	8.5 (5.1–11.9)	<0.001
**Summary scales**				
Physical component score	52.7 ± 20.6	65.5 ± 23.6	12.8 (8.9–16.5)	<0.001
Mental component score	48.7 ± 21.4	59.5 ± 23.3	10.8 (7.1–14.4)	<0.001

## Data Availability

This observational study is registered with ClinicalTrials.gov, number NCT02294825 (14 November 2014).

## References

[1] Bazot M., Daraï E. (2017). Diagnosis of deep endometriosis: Clinical examination, ultrasonography, magnetic resonance imaging, and other techniques. Fertil. Steril..

[2] Exacoustos C., Zupi E., Piccione E. (2017). Ultrasound Imaging for Ovarian and Deep Infiltrating Endometriosis. Semin. Reprod. Med..

[3] Berlanda N., Somigliana E., Frattaruolo M.P., Buggio L., Dridi D., Vercellini P. (2017). Surgery versus hormonal therapy for deep endometriosis: Is it a choice of the physician?. Eur. J. Obstet. Gynecol. Reprod. Biol..

[4] Perelló M.F., Martínez-Zamora M.Á., Torres X., Munrós J., Balasch Cortina J., Carmona F. (2017). Endometriotic Pain Is Associated with Adenomyosis but Not with the Compartments Affected by Deep Infiltrating Endometriosis. Gynecol. Obstet. Investig..

[5] Donnez O., Roman H. (2017). Choosing the right surgical technique for deep endometriosis: Shaving, disc excision, or bowel resection?. Fertil. Steril..

[6] Marinho M.C.P., Magalhaes T.F., Fernandes L.F.C., Augusto K.L., Brilhante A.V.M., Bezerra L.R.P.S. (2018). Quality of Life in Women with Endometriosis: An Integrative Review. J. Womens Health (Larchmt).

[7] Ware J.E., Sherbourne C.D. (1992). The MOS 36-item short-form health survey (SF-36). I. Conceptual framework and item selection. Med. Care.

[8] Bourdel N., Alves J., Pickering G., Ramilo I., Roman H., Canis M. (2015). Systematic review of endometriosis pain assessment: How to choose a scale?. Hum. Reprod. Update.

[9] Stull D.E., Wasiak R., Kreif N., Raluy M., Colligs A., Seitz C., Gerlinger C. (2014). Validation of the SF-36 in patients with endometriosis. Qual. Life Res..

[10] Eypasch E., Williams J.I., Wood-Dauphinee S., Ure B.M., Schmülling C., Neugebauer E., Troidl H. (1995). Gastrointestinal Quality of Life Index: Development, validation and application of a new instrument. Br. J. Surg..

[11] Agarwal B.B., Sharma S. (2012). Scoring Systems in Evaluation of Constipation and Obstructed Defecation Syndrome (ODS). J. Int. Med. Sci. Acad..

[12] Ware J.E., Gandek B. (1998). Methods for testing data quality, scaling assumptions, and reliability: The IQOLA Project approach. International Quality of Life Assessment. J. Clin. Epidemiol..

[13] Altman D.G., Bland J.M. (1997). Statistics notes: Cronbach’s alpha. BMJ.

[14] McHorney C.A., Ware J.E., Lu J.F., Sherbourne C.D. (1994). The MOS 36-item Short-Form Health Survey (SF-36): III. Tests of data quality, scaling assumptions, and reliability across diverse patient groups. Med. Care..

[15] Leplee A., Ecosse E., Pouchot J., Coste J., Perneger T. (2001). Le questionnaire MOS SF-36. Manuel de L’utilisateur et Guide D’interpretation des Scores.

[16] Bjorner J.B., Wallenstein G.V., Martin M.C., Lin P., Blaisdell-Gross B., Tak Piech C., Mody S.H. (2007). Interpreting score differences in the SF-36 Vitality scale: Using clinical conditions and functional outcomes to define the minimally important difference. Curr. Med. Res. Opin..

[17] Bourdel N., Chauvet P., Billone V., Douridas G., Fauconnier A., Gerbaud L., Canis M. (2019). Systematic review of quality of life measures in patients with endometriosis. PLoS ONE.

[18] Redwine D.B., Wright J.T. (2001). Laparoscopic treatment of complete obliteration of the cul-de-sac associated with endometriosis: Long-term follow-up of en bloc resection. Fertil. Steril..

[19] Dubernard G., Rouzier R., David-Montefiore E., Bazot M., Darai E. (2008). Use of the SF-36 questionnaire to predict quality-of-life improvement after laparoscopic colorectal resection for endometriosis. Hum. Reprod..

[20] Silveira da Cunha Araújo R., Abdalla Ayroza Ribeiro H.S., Sekula V.G., da Costa Porto B.T., Ayroza Galvão Ribeiro P.A. (2014). Long-term outcomes on quality of life in women submitted to laparoscopic treatment for bowel endometriosis. J. Minim. Invasive Gynecol..

[21] Touboul C., Ballester M., Dubernard G., Zilberman S., Thomin A., Daraï E. (2015). Long-term symptoms, quality of life, and fertility after colorectal resection for endometriosis: Extended analysis of a randomized controlled trial comparing laparoscopically assisted to open surgery. Surg. Endosc..

[22] Riiskjær M., Forman A., Kesmodel U.S., Andersen L.M., Ljungmann K., Seyer-Hansen M. (2018). Pelvic Pain and Quality of Life Before and After Laparoscopic Bowel Resection for Rectosigmoid Endometriosis: A Prospective, Observational Study. Dis. Colon Rectum.

[23] Roman H., Bubenheim M., Huet E., Bridoux V., Zacharopoulou C., Daraï E., Collinet P., Tuech J.J. (2018). Conservative surgery versus colorectal resection in deep endometriosis infiltrating the rectum: A randomized trial. Hum. Reprod..

[24] Garavaglia E., Inversetti A., Ferrari S., De Nardi P., Candiani M. (2018). Are symptoms after a colorectal segmental resection in deep endometriosis really improved? The point of view of women before and after surgery. J. Psychosom. Obstet. Gynaecol..

[25] de Freitas Fonseca M., Aragao L.C., Sessa F.V., Dutra de Resende J.A., Crispi C.P. (2018). Interrelationships among endometriosis-related pain symptoms and their effects on health-related quality of life: A sectional observational study. Obstet. Gynecol. Sci..

[26] Meuleman C., Tomassetti C., D’Hoore A., Van Cleynenbreugel B., Penninckx F., Vergote I., D’Hooghe T. (2011). Surgical treatment of deeply infiltrating endometriosis with colorectal involvement. Hum. Reprod. Update.

[27] Meuleman C., Tomassetti C., D’Hooghe T.M. (2012). Clinical outcome after laparoscopic radical excision of endometriosis and laparoscopic segmental bowel resection. Curr. Opin. Obstet. Gynecol..

[28] Brătilă E., Comandașu D., Coroleucă C., Cîrstoiu M., Bohîlţea R., Mehedinţu C., Vlădăreanu S., Berceanu C., Dan L.D. (2016). Gastrointestinal symptoms in endometriosis correlated with the disease stage. Proceedings of the 36th National Congress of Gastroenterology, Hepatology and Digestive Endoscopy.

[29] Roman H., Moatassim-Drissa S., Marty N., Milles M., Vallée A., Desnyder E., Stochino Loi E., Abo C. (2016). Rectal shaving for deep endometriosis infiltrating the rectum: A 5-year continuous retrospective series. Fertil. Steril..

[30] Roman H., Darwish B., Bridoux V., Chati R., Kermiche S., Coget J., Huet E., Tuech J.J. (2017). Functional outcomes after disc excision in deep endometriosis of the rectum using transanal staplers: A series of 111 consecutive patients. Fertil. Steril..

[31] Roman H., Ness J., Suciu N., Bridoux V., Gourcerol G., Leroi A.M., Tuech J.J., Ducrotté P., Savoye-Collet C., Savoye G. (2012). Are digestive symptoms in women presenting with pelvic endometriosis specific to lesion localizations? A preliminary prospective study. Hum. Reprod..

[32] Afors K., Centini G., Fernandes R., Murtada R., Zupi E., Akladios C., Wattiez A. (2016). Segmental and Discoid Resection are Preferential to Bowel Shaving for Medium-Term Symptomatic Relief in Patients With Bowel Endometriosis. J. Minim. Invasive Gynecol..

[33] Roman H., Loisel C., Resch B., Tuech J.J., Hochain P., Leroi A.M., Marpeau L. (2010). Delayed functional outcomes associated with surgical management of deep rectovaginal endometriosis with rectal involvement: Giving patients an informed choice. Hum. Reprod..

[34] Meuleman C., Tomassetti C., Wolthuis A., Van Cleynenbreugel B., Laenen A., Penninckx F., Vergote I., D’Hoore A., D’Hooghe T. (2014). Clinical outcome after radical excision of moderate-severe endometriosis with or without bowel resection and reanastomosis: A prospective cohort study. Ann. Surg..

[35] Bailly E., Margulies A.L., Letohic A., Fraleu-Louër B., Renouvel F., Panel P. (2013). Évolution des symptômes et de la qualité de vie des patientes après chirurgie de l’endométriose digestive [Evolution of symptoms and quality of life of patients after surgery of digestive endometriosis]. Gynecol. Obstet. Fertil..

[36] Lyons S.D., Chew S.S., Thomson A.J., Lenart M., Camaris C., Vancaillie T.G., Abbott J.A. (2006). Clinical and quality-of-life outcomes after fertility-sparing laparoscopic surgery with bowel resection for severe endometriosis. J. Minim. Invasive Gynecol..

